# Influence of Lamination Conditions of EVA Encapsulation on Photovoltaic Module Durability

**DOI:** 10.3390/ma16216945

**Published:** 2023-10-29

**Authors:** Dan Wu, Patrick Wessel, Jiang Zhu, Daniel Montiel-Chicharro, Thomas R. Betts, Anton Mordvinkin, Ralph Gottschalg

**Affiliations:** 1 Centre for Renewable Energy Systems Technology (CREST), School of Mechanical, Electrical and Manufacturing Engineering, Loughborough University, Loughborough LE11 3TU, UK; zhuj8331@hotmail.com (J.Z.); t.r.betts@lboro.ac.uk (T.R.B.); 2 Fraunhofer Center for Silicon-Photovoltaic (CSP), Otto-Eissfeldt-Straße 12, 06120 Halle, Germany; patrick.wessel@imws.fraunhofer.de (P.W.); anton.mordvinkin@csp.fraunhofer.de (A.M.); 3 Fachbereich Elektrotechnik, Maschinenbau und Wirtschaftsingenieurwesen (EMW), Hochschule Anhalt Bernburger Str. 57, 06366 Köthen, Germany; 4 Turbo Power Systems Ltd., 1 Queens Park, Queensway North, Gateshead, Tyne and Wear NE11 0QD, UK

**Keywords:** lamination conditions, curing level, EVA, reliability, durability, encapsulation

## Abstract

Encapsulation is a well-known impact factor on the durability of Photovoltaics (PV) modules. Currently there is a lack of understanding on the relationship between lamination process and module durability. In this paper, the effects of different lamination parameters on the encapsulant stability due to stress testing have been investigated from both on-site production quality and long-term stability viewpoints. Rather than focusing on single stability factors, this paper evaluates lamination stability using a number of indicators including EVA (ethylene-vinyl acetate copolymer) curing level, voids generation, chemical stability, optical stability, and adhesion strength. The influences of EVA curing level on the stability of other properties are also discussed. It is shown that laminates stability increases with increasing curing level to an upper limit, beyond which leading to the formation of voids, reduced transmittance stability, discoloration, and unstable interfaces. A minimum gel content is identified but an upper limit should not be surpassed. The best range of gel content for the materials tested here is 84–90%. Samples with gel content below 70% show low chemical and optical stability, weak adhesion strength, and EVA flowing. Laminates with gel content over 92% are more likely to become yellow and are less stable in adhesion.

## 1. Introduction

Crystalline silicon photovoltaic (c-Si PV) modules require encapsulation for the protection of the active elements from the environment. This is achieved with a multilayer system with high weatherability by laminating a stack of glass–encapsulant–active layer–encapsulant–backsheet with controlled temperature, pressure, and duration [1,2,3,4]. As will be shown later, different lamination ‘recipes’ (of varying the process control parameters above) show different reliability levels and will influence the long-term field performance. As an example, different levels of power degradation have been reported for EVA (ethylene-vinyl acetate copolymer) laminated modules cured at different temperatures [5]. Optimisation of lamination is often carried out in terms of production throughput and passing basic quality requirements. The initial requirements are to pass qualification testing of the IEC (International Electrotechnical Commission) -61215 standard, which is designed to identify early known failures [6]. However, optimisation of the lamination conditions is a complex issue and should be evaluated not limited to passing qualification testing, but to ensure long-term performance and durability. There are several published studies on lamination conditions, but they only focus on a single material property, e.g., optical transmission, crosslinking degree, or adhesion [7,8,9,10,11,12,13]. In reality, these different properties are correlated and need to be assessed concurrently. It is likely enhancing one property may deteriorate another at the same time. In addition, the specific properties are predominantly assessed on ‘as produced’ devices rather than devices under stress testing. The resistance of different properties to various environmental stresses is key for longevity [14]. A parametric approach investigating the influence of lamination conditions on the encapsulation durability is developed in this paper.

The current market dominating encapsulant is EVA [15]. During the encapsulation of PV modules with EVA, two of the important material changes are the curing reaction leading to material cross-linking and interfacial adhesion formation. The cross-linking degree and adhesion strength is commonly checked for quality control.

Cross-linking degree can be determined by several different methodologies, ranging from Soxhlet, Raman spectroscopy, differential scanning calorimetry (DSC), rheology, or thermo-mechanical indentation etc. [16,17,18,19,20]. Several of these methods have been included in the IEC 62788-1-6 [21]. Soxhlet method is seen as the primary method which measures gel content from which curing level is calculated. Curing level recommendations are manufacturer dependent, e.g., minimum gel contents range from 60% to 90%. Manufacturers tend to specify minimum levels only. This originates in research by the Jet Propulsion Laboratory (JPL), who found that the main requirement for passing the certification tests is a minimum gel content of 65% [22]. It is not certain that this is met in today’s production. PI Berlin conducted a field test measuring the gel content of 254 EVA samples extracted from 120 PV modules and showed that only two thirds of the tested EVA samples had an appropriate gel content [23]. It is shown in this study that there is also an upper limit beyond which durability will deteriorate. Various properties of PV devices are affected by curing levels of the encapsulant, including chemical, mechanical, and optical stability. It remains to be demonstrated how curing levels affect overall encapsulation performance in terms of durability and the required data for one material system is presented in this study.

For multi-layer encapsulation system, a minimum adhesion is required to prevent delamination and thus maintain mechanical integrity of the package. STR (a manufacturer of EVA) suggests a minimum of 53 N/cm peel strength after lamination (measured by 180° peel) to be sufficient to ensure a 20-year life-time [24]. Pern and Glick [25] examined the adhesion strength between glass and EVA for samples with various backsheets and EVA composition through 90° peel test. The reported peel strengths were in the range of 10 N/cm to 120 N/cm, with no valuation given for what is sufficient. Tracy et al. [26] developed a new adhesion metrology using width-tapered cantilever beam to measure the debonding energy. Initial threshold values to avoid delamination were proposed to be 160 J/m^2^ for encapsulant interfaces and 10 J/m^2^ for backsheet interface. Dadaniya and Datla [27] developed a numerical model to predict the adhesion strength degradation at encapsulant-glass interface under stress dose. There are different adhesion mechanisms at play. The adhesion between glass and EVA derives mainly from the silicon-oxygen covalent bonds formed between glass and the silane coupling agents within EVA. The adhesion between EVA and backsheet are due to the mutual diffusion between EVA and the EVA compatible material at the inner side of the backsheet [10,28,29]. Both of these two mechanisms are influenced by lamination processes. As can be seen, adhesion requirements for PV modules to ensure long-term reliability has been studied but not well defined due to the complexity of testing methods, material differences, and stress variation etc. Therefore, there is a lack of standard value or minimum value of adhesion strength that guarantees optimal performance after stress test or after long-term operation in real outdoor conditions. The lack of such standard evaluation has caused lots of limitations such as quality control, regulatory compliance, cost control and reliability, and durability etc. The study of the adhesion strength in this paper will contribute to the understanding of the adhesion behaviour and lamination conditions.

Through indoor stress tests, this paper investigates the dependence of long-term durability of the encapsulation systems on lamination conditions from various aspects considering void generation, curing level, chemical, and optical stability as well as the adhesion strength at the glass-EVA interface and the EVA-backsheet interface. The influence of curing degree of EVA on the reliability of the encapsulation system is also discussed. Optimal lamination condition is assessed for the tested samples considering the factors mentioned above.

## 2. Experimental Design

**Figure 1:**
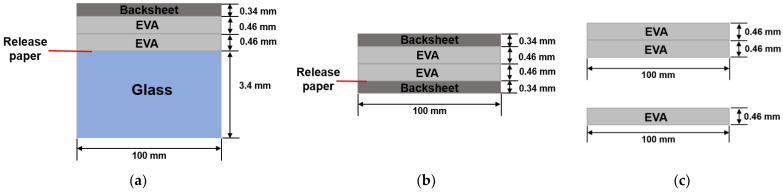
Sample configurations: (**a**) standard configuration, but without a solar cell and with a release paper, (**b**) EVA and backsheet with a release paper and (**c**) double layer EVA (upper) and single layer EVA (lower) without a release paper.

This paper focuses on the degradation and stability behaviour of PV laminates through the study of laminated samples without cells as shown in Figure 1. Samples were laminated at eight different conditions and then were subjected to indoor ageing tests in environmental chambers. Standard damp-heat (85 °C-85% RH) and thermal cycling (−40–85 °C) were conducted according to the IEC 61215 standard [6]. Mechanical, optical, and chemical properties of laminates were measured and studied over the course of ageing.

The encapsulant material is a fast-curing EVA from EVASA in Spain with a curing agent of Lupersol TBEC, a vinyl acetate content of 34%, and a thickness approximately 0.46 mm. The backsheet is a multilayer polymer with a structure of polyethylene terephthalate (PET)-PET-EVA, of overall thickness 0.34 mm from Dupont Teijin Films. Low iron, un-tempered float glass from Saint-Gobain (Leicestershire, UK) with a thickness of 3.4 mm is used in all glass/EVA-EVA/backsheet laminates. The glass was submerged in deionised water for 20 min, cleaned with isopropanol solution and dried before lamination.

**Figure 2:**
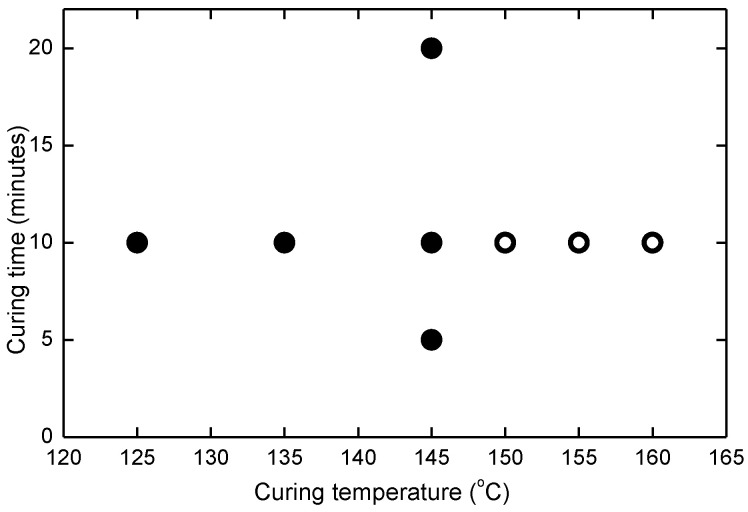
Lamination conditions applied to each sample configuration (solid circles indicate conditions lead to no voids in the laminate, hollow circles indicate conditions leading to voids and very low adhesion strength).

Samples were laminated at eight conditions using a 2BG L176A laminator (Figure 2). The vacuum time is 5 min and the lamination pressure is 100 kPa. Two conditions with the curing temperature of 155 °C and 160 °C for 10 min lead to large amount of bubbles and one condition of 150 °C curing temperature for 10 min curing time has very low initial adhesion, which were excluded in further study (hollow marks in Figure 2). Thus, five conditions which lead to no voids were selected (solid marks in Figure 2). This includes three different curing temperatures of 125 °C, 135 °C and 145 °C with a fixed curing time of 10 min as well as curing times of 5, and 20 min at the curing temperature of 145 °C.

To measure the adhesion strength at the glass-EVA interface (GEI), laminate samples of 100 × 150 mm size with glass/EVA/EVA/backsheet structure were produced (Figure 1a). The adhesion strength was measured by 90° peel test with a crosshead speed of 50 mm/min at ambient temperature. A CO_2_ (carbon dioxide) laser system was used to cut the backsheet together with EVA into 10 mm wide strips for peel testing [30]. After cutting, eight strips were produced for each sample. The reliability of the adhesion strength at this interface was checked through both damp-heat and thermal cycling tests. At each testing point, 24 strips (less if it breaks during testing) from three laminates were peeled and their average peel strength was calculated. In order to analyse the chemical changes of EVA during ageing tests, nine strips that were peeled off from three laminates were randomly selected each time during damp-heat exposure. They were analysed by FTIR-ATR (fourier transform infrared-attenuated total reflectance) in the spectra range of 4000 cm^−1^ to 600 cm^−1^ using a Perkin Elmer Spectrum One FTIR-ATR machine. The resolution of the scan is set to 4 cm^−1^ and the crystal used in the ATR is Diamond/ZnSe.

Adhesion strength at the EVA-backsheet interface (EBI) was measured through T-peel test using 100 × 150 mm sized samples of backsheet/EVA/EVA/backsheet (Figure 1b). The samples were cut into 10 mm wide strips with a sharp knife. T-peel test was conducted at ambient temperature with a crosshead speed of 50 mm/min. Six strips were measured for each lamination condition and the average was calculated.

Six 10 × 10 cm free-standing EVA sheets (Figure 1c) were also cured at each lamination condition. Three of them were single layer EVA used to test cross-linking degree through solvent extraction method with xylene as the extraction solvent [31]. The other three were double layer EVA used for transmission measurement with a Cary 5000 spectrophotometer (Agilent Technologies, Santa Clara, CA, USA) in the spectral range of 200–1200 nm with 1 nm resolution and an average integrating time of 0.1 s. The light source of the spectrophotometer is a tungsten halogen lamp with a correlated colour temperature approximating CIE standard illuminant A. Transmittance was tested at two randomly selected locations for each sample at each testing point. Ideally, both crosslinking and transmittance tests should be conducted on EVA extracted from the laminates after peel test. However, the stress during the extraction of EVA may change EVA crystallinity by exerting orientation upon the molecular chain. The deformation of EVA will also influence the results of transmittance measurements. Thus, free standing samples were used. This is to represent the worst case in terms of moisture ingress. The yellowing index (YI) was then calculated according to the standard ASTM (American Society for Testing and Materials) E313 based on the measured transmittance results [32]:

YI = 100 × (C_X_ × X − C_Z_ × Z)/Y (1)

where X, Y, Z are tristimulus values of the measured object; C_X_, and C_Z_ are numerical coefficients used for calculation and their values depend on the types of standard illuminant and observer. CIE (International Commission on Illumination) standard illuminant D65 is used for the calculation according to ISO (International Organisation for Standardisation)/CIE 11664-2 [33]. CIE 1931 standard colorimetric system is chosen so that the observer is the CIE 1931 standard colorimetric observer whose color-matching properties correspond to the CIE 1931 color-matching functions [34]. The calculation of the tristimulus values is based on ISO/CIE 11664-3 [35].

**Figure 3:**
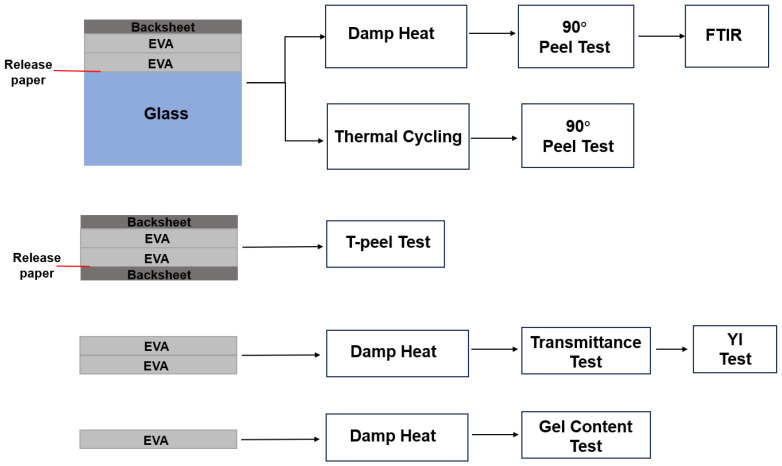
Flowchart of the experimental plan.

A flowchart of the overall experimental plan is depicted in Figure 3.

## 3. Dependence of the Stability of Encapsulation Systems on Lamination Conditions

### 3.1. Gel Content

**Figure 4:**
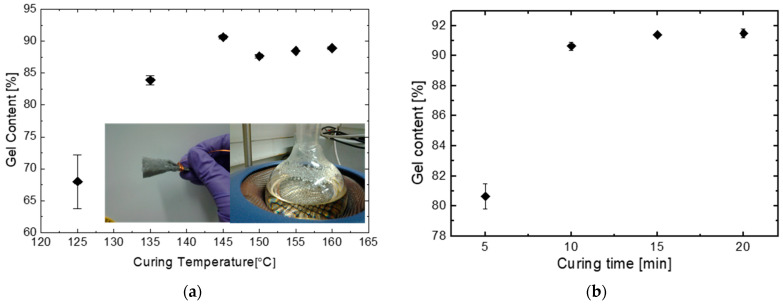
Gel content of EVA cured at different conditions: (**a**) 10 min curing time at different curing temperature (inserted photos showing gel leakage at T125M10); (**b**) 145 °C curing temperature and different curing time (T: curing temperature in °C, M: curing time in min).

EVA gel content is measured by Soxhlet method and the results are shown in Figure 4. The expanded relative uncertainty of the gel content measurement in this paper is around ±0.9% (k = 2) based on the ISO guide to the expression of uncertainty in measurement (GUM) [36,37]. For curing time of 10 min, gel content increases as the curing temperature increases from 125 °C to 145 °C and then decreases with further curing temperature increase. The decrease over 145 °C may be because too high temperature causes the decomposition of peroxide. Oxygen will be generated when peroxide decomposes and this will cause bubbles in the laminates (see results in Section 3.2). For fixed temperature, the gel content also increases quickly from 80% to 90% with the increasing curing time from 5 min to 10 min and then the increase slows down until it stabilises at 92%.

As seen in the inserted photo in Figure 4a, it is identified that for EVA cured at 125 °C with a curing time of 10 min, denoted by T125M10 with T representing the curing temperature in °C and M indicating the curing time in minutes (same as the other conditions), small sticky particles in a molten state were identified on the surface of the flask and the mesh. This indicates that gel leaked out and thus is a sign of EVA not being well cured. The standard deviation of the measurements is noticeable larger at this condition because of the gel leakage. The leakage of the poor cross-linked low molecular weight fractions is counted into the soluble parts, even though it should be part of the crosslinked fraction. The identification of gel leakage further highlights that samples cured under T125M10 is not suitable to use xylene extraction method for crosslinking degree measurement. As a result, the outcomes presented here for this condition are primarily to offer an indication of the potential crosslinking degree rather than a precise measurement.

**Figure 5:**
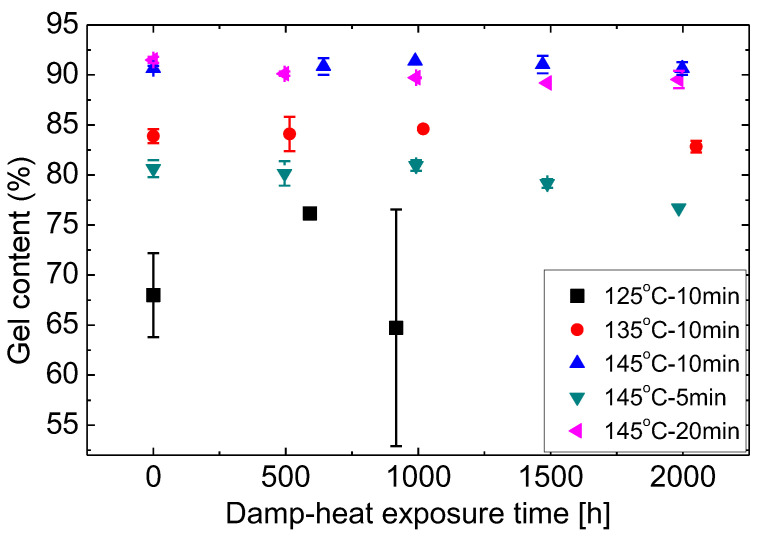
Changes of gel content during damp-heat exposure.

To see the effect of long-term exposure to humidity and temperature on the gel content, samples of EVA sheets were subjected to damp-heat exposure at the standard condition of 85 °C/85% relative humidity. Gel content measurements were conducted at different time points as shown in Figure 5. No observable changes are identified for samples cured at T135M10, T145M10, and T145M20 with gel content in the order of 85–90%. A slight reduction at T145M5 is seen which may be due to material non-uniformity and experimental uncertainty. Thus, no further curing due to damp-heat exposure can be identified for these gel content levels. Gel leakage is also observed for the T125M10 samples even after 2000 h damp-heat exposure which introduced large variability in the results.

### 3.2. Voids Formation

**Figure 6:**
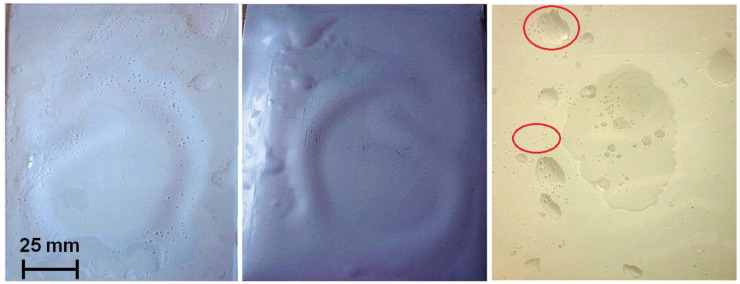
Voids formed at the curing condition of T160M10.

Void free is a critical criterion for module reliability. Laminates produced with higher curing temperatures (T160M10, T155M10 and T150M10) exhibited voids. Figure 6 depicts typical voids of T160M10 samples. There are two different types of voids. The first type is at the EVA-backsheet interface (EBI). Void size is relatively large. Interconnections are often observed for this case, e.g., at a temperature of 160 °C. The co-joined area covers a significant percentage of the laminates, potentially leading to delamination. The second type of voids is typically spherical in shape. These bubbles occur within the EVA layer. Sizes are much smaller than that for EBI voids, with diameters typically below 2 mm. A consequence of this second type is a loss of adhesion at glass-EVA interface (GEI) and EVA-backsheet interface (EBI). These two types of bubbles have been reported and investigated in a broad range of studies [38,39,40,41].

Reducing the curing temperature to 155 °C result in type I voids free samples with only few small bubbles trapped within the EVA. A further reduction of curing temperature to 150 °C yields void-free laminates with almost no bubbles trapped within EVA.

### 3.3. Chemical Stability of EVA Cured at Different Conditions

**Figure 7:**
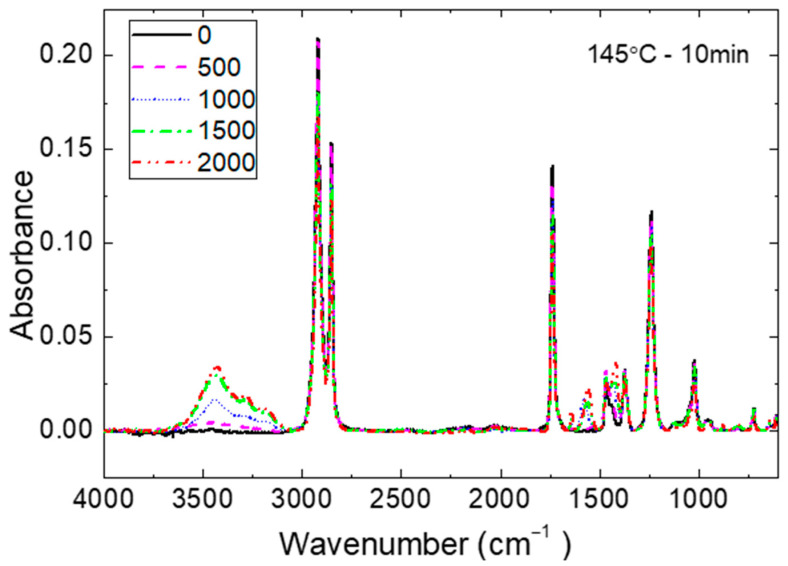
Changes of FTIR-ATR spectrum of T145M10 sample during damp-heat test.

FTIR-ATR spectrum of EVA throughout damp-heat (DH) stressing are shown in Figure 7 for the sample T145M10. The absorption of hydroxyl peaks in the range of 3200–3800 cm^−1^ increases with the increasing exposure time. The potential causes could be either of these explanations:

- moisture ingress manifesting as hydrogen bonded to the acetate groups;
- vinyl acetate hydrolysis generating a molecular hydroxyl group and acetic acid;
- oxidation of the encapsulant.

With the increasing of DH exposure time, three peaks decrease which are the acetate C=O peak (1735 cm^−1^), the ester C-O peak (1236 cm^−1^), and C-O-C peak (1018 cm^−1^). This agrees well with the progressing hydrolysis of EVA reported in [42,43,44]. The peaks at 2950 cm^−1^ and 2918 cm^−1^, denoting symmetrical and asymmetrical stretching of methylene in the backbone of EVA, respectively, also decrease during exposure. The most probable cause is the Norrish reaction, which can generate polyenes and unsaturated carbonyls to cause EVA discoloration [45]. Besides the degradation of EVA, additives within EVA are also a source of discoloration [46,47,48,49]. The discoloration rates of EVA with different additive formulations were examined by Peike et al. [46]. EVA with combined additives showed larger discoloration than EVA with a single additive, independent of its nature (crosslinking agent, an UV absorber, an UV stabiliser, or an antioxidant). Klemchuk et al. [49] found that EVA discoloration is most likely due to additive interactions, notably peroxide-UV absorber and peroxide-phosphate. It is noticed in Figure 7 that two new peaks at about 1560 cm^−1^ and 1650 cm^−1^ are observed after exposure. The peak at 1560 cm^−1^ is attributed to methylene group near the easter group while the peak at around 1650 cm^−1^ could be attributed to conjugated carbonyl or C=C vibration [50,51,52,53].

**Figure 8:**
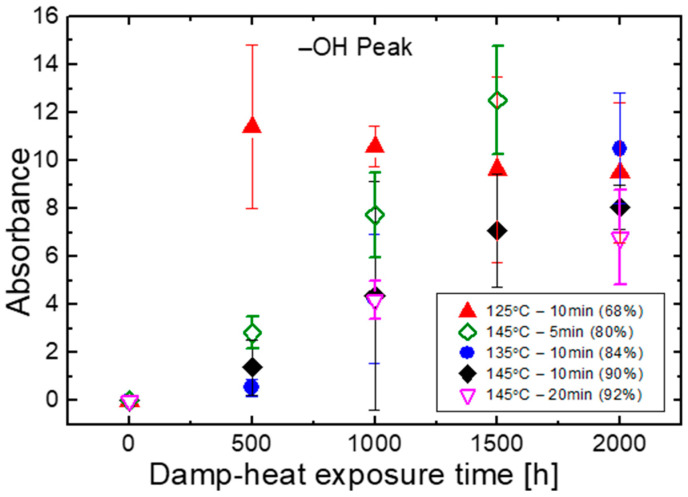
Changes of ATR absorbance of hydroxyl groups during damp-heat exposure.

**Figure 9:**
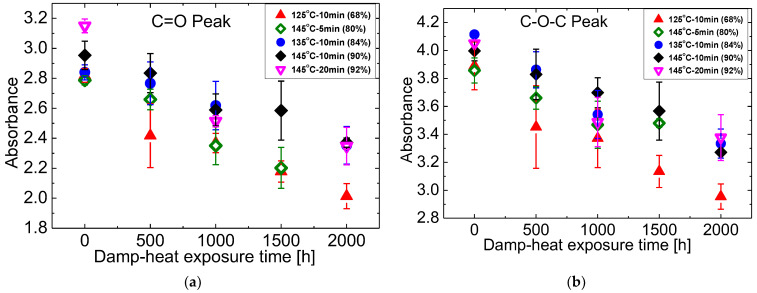
Changes of ATR absorbance at (**a**) 1735 cm^−1^ and (**b**) 1236 cm^−1^ during damp-heat exposure.

**Figure 10:**
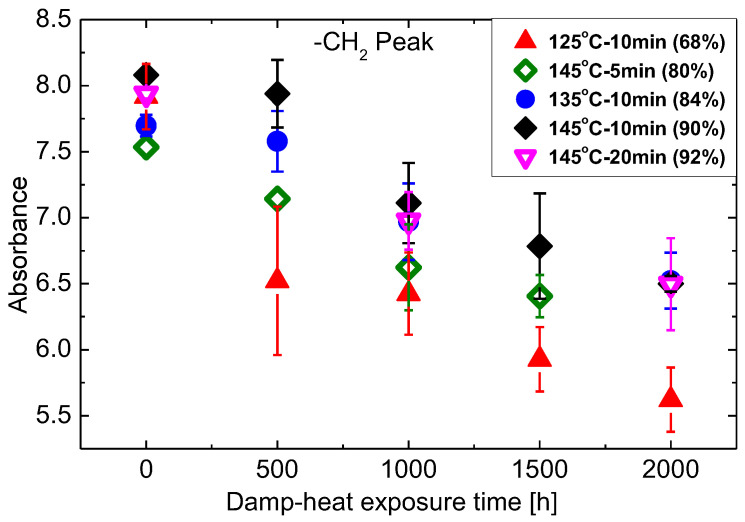
Changes of ATR absorbance at 2918 cm^−1^ during damp-heat exposure.

The integral under the peaks of absorbance allows an estimation of the changes during DH exposure. The results are shown in Figure 8, Figure 9 and Figure 10 as a basis to rank the chemical stability of EVA samples cured at the different conditions. The focus is on the hydroxyl groups (3800–3200 cm^−1^), ester groups (1735 cm^−1^ and 1236 cm^−1^), and the methylene group (2918 cm^−1^).

The hydroxyl peak (3800–3200 cm^−1^) reflects the stability of EVA to resist moisture ingress and oxidation. T125M10 samples, i.e., the group with the lowest gel content of 68%, shows the largest and the quickest increase of this particular peak. This peak increases sharply from 0 h to 500 h and maintains at the level till 2000 h. T145M5, with gel content of 80%, exhibits the next most significant change. T135M10, T145M10 and T145M20, with gel contents higher than 84%, saw similar increases with a lower rate during the first 500 h and a higher rate thereafter. They reach the same level where the T125M10 samples are after 2000 h. The standard deviation of the absorbance result becomes larger as the peak area increases. This is due to the uneven degradation throughout the whole sample. Higher gel content results in higher chemical stable material than lower gel content does.

Figure 9 quantifies EVA hydrolysis utilising the two peaks at 1735 cm^−1^ and 1236 cm^−1^. It demonstrates the similar dependence on gel content with the hydroxyl group. EVA with the lowest gel content of 68% that cured at T125M10 sees the largest and fastest decline, followed by the samples with the gel content of 80% that prepared at T145M5. There are no significant differences between the other three EVA with the gel content higher than 84%. Figure 10 which depicts the changes of the methylene groups during damp heat exposure also follows the same trend.

Curing levels seem to correlate with chemical stability: increasing gel content results in more chemically resilient materials until around 84% gel contents where no obvious improvement can be seen. Different EVAs will behave slightly differently, but for the given EVA tested in the work, gel contents above a threshold of 84% ensure good stability. Gel contents below 70% are prone to chemical degradation such as oxidation, hydrolysis, and backbone breakage.

### 3.4. Optical Stability of EVA Cured at Different Conditions

**Figure 11:**
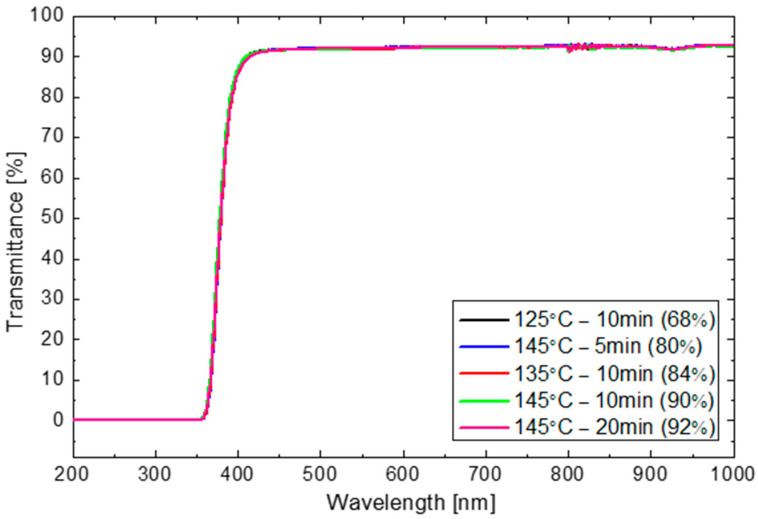
Transmittance of EVA cured at different conditions.

There is no apparent difference in transmittance between the as-produced samples at different conditions as shown in Figure 11. The transmittance in the visible range is about 93%. The transmittance is negligible for wavelengths below 360 nm where the UV absorber blocks transmission.

**Figure 12:**
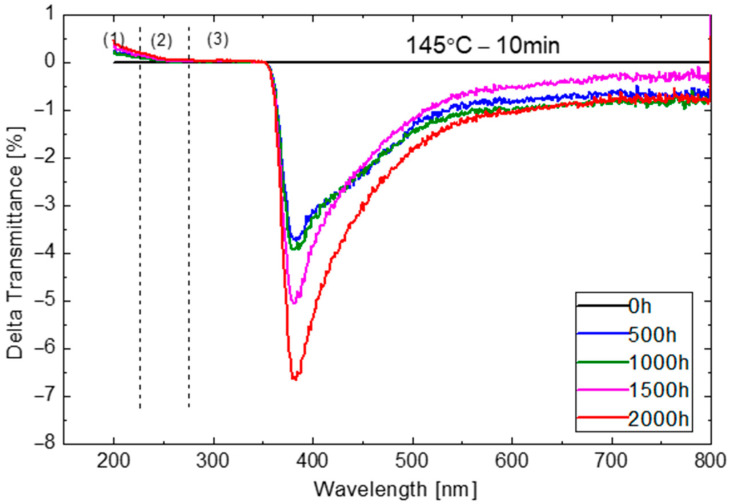
Net transmittance changes of EVA cured at T145M10 during damp-heat exposure.

Damp-heat exposure has an influence on the transmission of EVA, as plotted in Figure 12 exemplarily shown for the T145M10 sample. Three regions are used for further analysis: 200–270 nm, 270–360 nm and 360–800 nm, for sake of simplicity termed region (1), (2) and (3) in the following. Region (1) exhibits a slight improvement in transmission, albeit this could be within the realms of measurement uncertainty. Another possible explanation is a secondary effect of chromophores being formed due to humidity ingress into the laminates. Region (2) is stable throughout the stress exposure. The UV absorber is strongly absorbing in this spectral range. This would indicate no, or very limited, depletion of the UV absorber within EVA. Region (3) exhibits spectrally non-uniform loss in transmission. This may be due to the chromophore formation due to chemical interaction with water. Another reason could be light scattering caused by absorbed moisture [54], although the relatively pronounced peak of transmission loss at around 380 nm would suggest chromophores as a more likely explanation. This area from 380 nm to 500 nm corresponds to specific colours being absorbed, i.e., purple and blue light. This results in mixing green and red light, causing ‘yellowing’ in the EVA.

**Figure 13:**
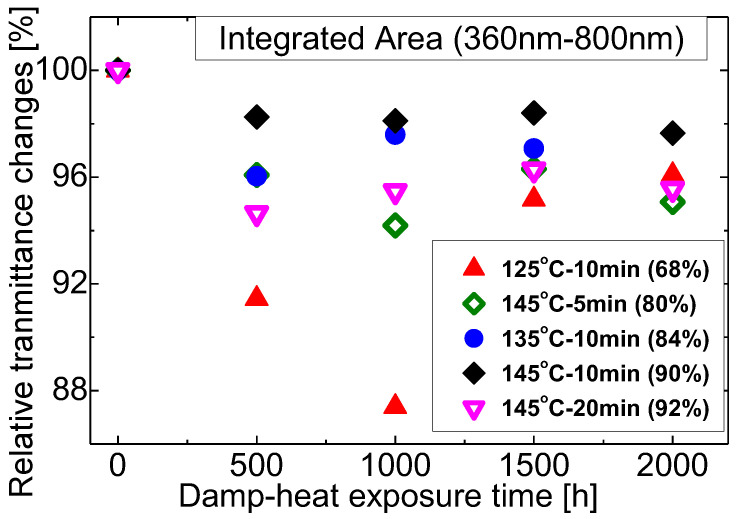
Relative change of the integrated transmittance between 360–800 nm vs. exposure time.

The changes of the spectrum’s transmittance integrated in region (3) of 360–800 nm observed during damp-heat exposure for different lamination conditions are shown in Figure 13. Relative changes of integrated spectrum transmittance with that before exposure are plotted against the exposure time. T145M10 samples (gel content of 90%) sees about 2% reduction after 2000 h damp-heat exposure. This is the most stable set of samples in terms of optical stability. This is followed by EVA cured at T135M10 (gel content of 84%) and then T145M5 (gel content of 80%) where maximum reduction is about 4% and 6% at 500 h respectively. The largest optical degradation is seen by EVA cured at T125M10 which has the lowest gel content. The integrated transmittance degraded by 13% continuously during the first 1000 h of exposure and then recovers about 10% till 2000 h of exposure. The recovery of optical transmission during damp-heat exposure is seen by some samples. This unexpected improvement may be caused by oxidation of the generated chromophores at later stages of the degradation or because of the reduced moisture amount and reduced crystallinity. With the increasing of gel content from 68% to 90%, the transmittance loss reduces. However, increasing the gel content further does not improve optical stability but shows adverse effects. EVA with the highest gel content of 92% (cured at T145M20) shows comparable degradation with that of gel content of 80% (cured at T145M5). Although similar transmittance is achieved for the laminates cured at the five conditions on production as seen in Figure 11, large differences are demonstrated for the performance under damp-heat exposure. The largest transmittance differences can be higher than 10% at the exposure time of 1000 h while the smallest transmittance differences are around 2% at the exposure time of 2000 h.

**Figure 14:**
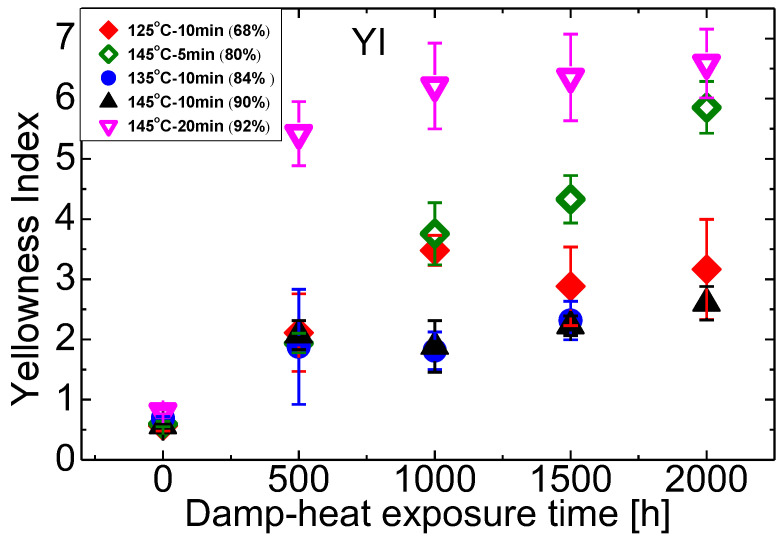
Changes of EVA’s yellowness index (YI) during damp-heat exposure.

EVA may become yellow when exposed to humidity under high temperatures. A measure of yellowness is the yellowness index (YI). YI indicates the degree of the material colour varies from a white standard towards yellow. YI changes of the various samples in this work are shown in Figure 14. Samples with gel contents around 85–90% are the most stable. The samples with the lowest gel content appear to be as stable as the best ones. This may be attributed to the oxidation as discussed earlier. Sample with the highest gel content sees the highest YI. Based on YI alone, one should strive for gel contents in the range of 85–90%.

### 3.5. Stability of the Adhesion Strength within the Encapsulation System Cured at Different Conditions

#### 3.5.1. Peel Strength of Laminates Cured at Different Conditions

**Figure 15:**
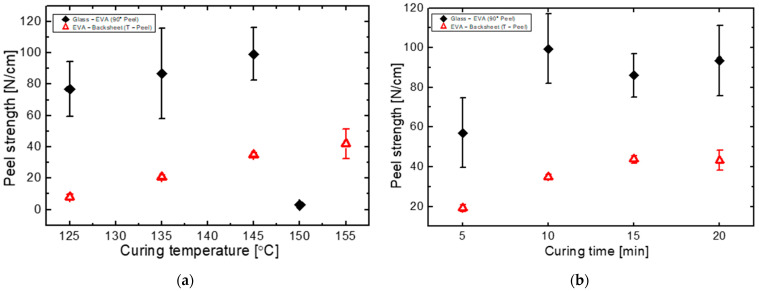
Peel strength measured at glass-EVA interface and EVA-backsheet interface for laminates cured at: (**a**) different curing temperature and 10 min curing time; (**b**) different curing time and 145 °C curing temperature.

Devices may fail at different interfaces, glass-EVA interface (GEI), or EVA-backsheet interface (EBI). The measured peel strengths at these interfaces in dependence of lamination condition are shown in Figure 15. Adhesion of GEI varies significantly but no statistically significant differences are identifiable at different curing temperatures. For samples cured at different times, T145M5 have the lowest adhesion and no differences are observed for curing time longer than 10 min. Adhesion strength at GEI is approximately 80–100 N/cm. The adhesion at this interface typically depends on silane coupling agents. Their action starts at relatively low temperatures and thus may less depend on lamination temperature than the lamination time. To obtain good adhesion at GEI, enough lamination time needs to be ensured. When peel the samples at T125M10, failure is more likely to happen at EBI which indicates the adhesion strength at EBI is much lower than that at GEI for this lamination condition.

Bubbles start to appear at T150M10. This influences GEI as adhesion is generally low. Adhesion is typically lower than 5 N/cm and EVA can be peeled off manually. Apparently, too fast crosslinking adversely affects adhesion. In the lamination system used here, curing temperatures in excess 145 °C are found too high. Samples cured at these conditions have such a low adhesion that stress testing is not feasible.

Adhesion at the EBI shows a clearer trend than GEI. Adhesion increases with the increasing curing temperature and the increasing curing time. The peel strength increases from approximately 7 N/cm to 45 N/cm while the temperature rises from 125 °C to 155 °C. When the curing time increases from 5 min to 15 min, the peel strength also increases from approximately 19 N/cm to 45 N/cm and stabilises thereafter. The difference of the GEI and EBI can be explained by the different adhesion mechanisms. Adhesion at EBI is determined by mutual diffusion of EVA and the EVA compatible film at the inner side of the backsheet. The diffusion increases with increasing temperature and contact time. As the curing level is positively correlated to curing temperature and time, lower curing levels are normally accompanied with lower adhesion strength at EBI.

The sensitivity of adhesion strength at EBI on curing conditions is further approved by the variation of failure interfaces during peel test of glass-EVA-backsheet laminates. The failure locus of 90° peel tests correlates gel content. The peel-locus shifts from EBI to mixed EBI and GEI and finally to GEI with rising gel content. This is due to increasing curing levels enhancing mutual diffusion of the bulk EVA and the EVA compatible materials (adhesion promoters) in the inner side of the backsheet. Thus, adhesion strength at EBI increases with increasing gel content until is significantly higher than that at GEI.

#### 3.5.2. Changes of Peel Strength at Glass-EVA Interface during Damp-Heat Exposure

**Figure 16:**
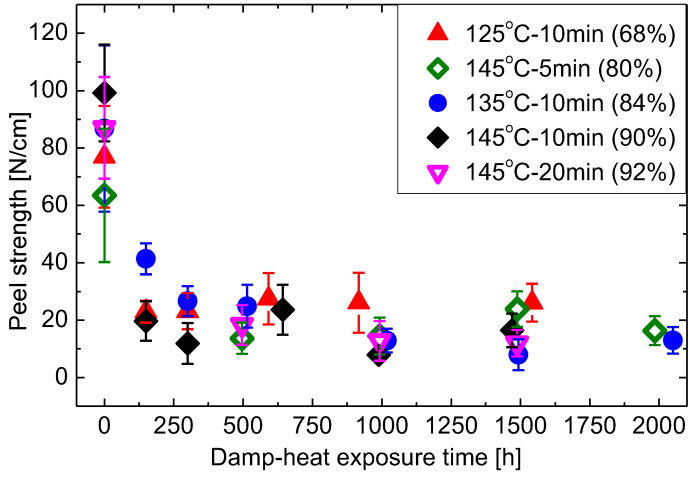
Changes of peel strength at glass-EVA interface during damp-heat exposure.

Although the peel strength at GEI initially varies slightly between the different fabrication conditions, not too much differences are observed with progressing exposure to damp heat, as shown in Figure 16. The shapes of the deterioration observed for all the samples are similar. Initial peel strengths vary between 60 and 100 N/cm. All samples then degrade quite quickly, within 300 h, to approximately 20 N/cm. Beyond this all samples remain fairly constant and fluctuates around 10–30 N/cm. T125M10 maintains the highest peel strength. The relatively low gel content causes EVA to remain rather viscous. At temperatures of 85 °C seen during damp heat exposure, EVA in these samples soften to a viscous melt. This wets the glass to re-build or build-up additional secondary bonds, causing improved adhesion strength. This, however, comes at the cost of a higher likelihood of cells shifting in the package and thus is not desirable. As can be seen from Figure 16, the standard damp-heat test of 85°C/85% RH is a relatively fierce condition for the stability of the adhesion strength of PV modules.

It is impossible to verify peel strength for T125M10, T145M10, and T145M20 samples beyond 1500 h of damp-heat exposure as the peel strips snapped during peel testing. The cohesive strength of the peel-strip reduced to below the adhesion strength. The strip snap initiated at outer PET (polyethylene terephthalate) layer of the backsheet and progressed to the other two layers as peeling continued. This is due to embrittlement of PET due to hydrolysis studied in e.g., [55,56]. Kempe et. al. [56] modelled PET hydrolysis in different locations and compared the resulting level of hydrolysis over 20 years of operation with damp-heat stressing at 85 °C/85% RH. It was found that 85 °C/85% RH is a too large stress and thus PET hydrolysis will seldom happen in outdoor conditions.

#### 3.5.3. Changes of Peel Strength at Glass-EVA Interface during Thermal Cycling

The mechanisms causing degradation of interfacial adhesion strength under cyclic thermal stresses are different to those of the reduction in damp-heat. Under thermal cycling, thermal stress is accumulated due to various factors such as mismatched thermal expansion coefficients (CTE) of the different components, shrinkage of adhesive in curing, trapped gases and differences in thermal conductivity [57,58]. After thermal cycling, the thermal stresses will result in a net residual stress at the glass surface which will cause reduced shear strength and unstable interface.

**Figure 17:**
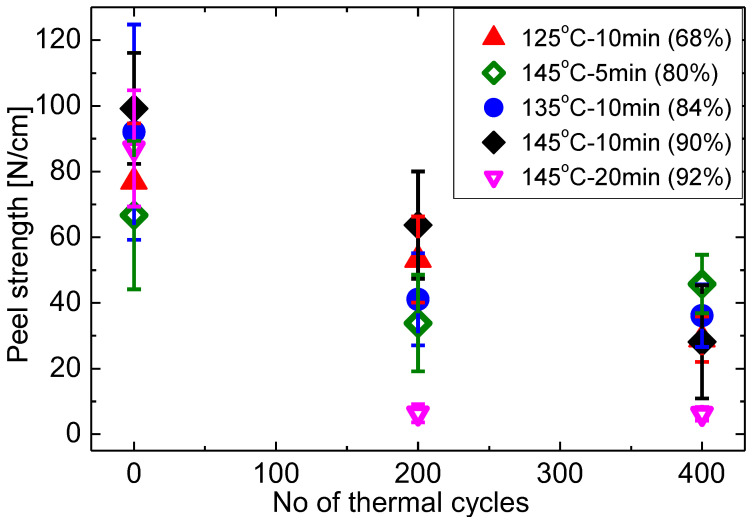
Changes of peel strength at GEI with progressing thermal cycling.

Standard thermal cycling (−40–85 °C) tests were conducted according to the IEC 61215. It is seen that peel strength at GEI deteriorates with increasing number of thermal cycles as shown in Figure 17. T145M20 laminates, the samples with the highest gel content and a high initial adhesion strength, demonstrates the lowest stability. After 200 thermal cycles both type 1 and type 2 bubbles appear, causing a drop-in adhesion. The occurrence of debonding is more likely due to the fact that higher curing levels result in shrinkage of EVA, which causes more residual thermal stresses within EVA during the cooling process at the end of lamination [59]. Li et al. [9] has experimentally detected this correlation between residual stresses and curing degrees of EVA. There are other possible reasons that high gel content leads to high stiffness. This increases the likelihood of cracks developing at the surface and results in reduced shear strength. Detailed mechanistically investigations are required for a conclusive explanation though, only empirical result is presented here.

T125M10, T135M10, T145M10, and T145M5 behave similarly in terms of peel strength reduction. T125M10 shows the least peel strength decrease followed by T145M10 and then T145M5 and T135M10 within 200 cycles. Incompletely cured samples will accumulate fewer residual stresses due to their viscosity. Unsurprisingly, T125M10 samples exhibit the least degradation within 200 cycles. After 400 cycles the peel strength of all samples degrades to the same level of around 30 N/cm ± 15 N/cm.

## 4. Optimum Lamination Condition

The stability of different properties of laminates cured at various conditions are summarised in Table 1. The damp-heat test results are showcased after 1000 h exposure while thermal cycling results are exemplified using data after 200 thermal cycles. A correlation between EVA curing degree (demonstrated by gel content) and lamination quality is observed, although this may only be a secondary correlation. The stability of chemical properties of EVA improves as the curing degree increases to a certain level over which no obvious improvements are seen. Different levels of optical stability are shown at different lamination conditions with the same as-produced property. The optical stability of EVA increase as the curing degree increases to a critical value over which the stability decreases. Adhesion strength at the EVA-backsheet interface is also observed to increase with the increasing curing degree. Initial adhesion strength at the glass-encapsulant interface is not significantly influenced by curing degrees but curing time. Enough curing time should be ensured to avoid low adhesion strength. Adhesion stability at this interface performs the best at the lowest gel contents which were not well cured under both damp-heat and thermal cycling stresses. It maintains similar stability with the increasing curing level to an upper limit after which the thermal stability decreases. In general, the quality of laminates increases with the increasing curing level to an upper limit beyond which the stability of laminates decreases. For the material system tested in this study, EVA with gel contents between 84% (cured at 135 °C for 10 min)–90% (cured at 145 °C for 10 min) exhibits comparable chemical, optical and adhesion stability and can be regarded as the optimal curing condition.

Gel contents below 70% have elevated risks of chemical degradation, reduced transmittance stability and lower adhesion at EBI. It, however, appears to exhibit the best GEI adhesion stability in both humid and thermal-cycling conditions. EVA melting and flowing allows the formation of strong secondary forces, which potentially introduces the risk of moving cells and detached cracks. Thus, this is not a desirable lamination condition. The given investigation indicates that low gel contents should be avoided, thus the previously proposed minimum gel content of 65% suggested by JPL is too low.

Too high curing degrees are also not desirable which will cause samples with variable optical properties. Potentially, this may enhance yellowing. Samples with too high curing degree also tend to result in less thermal stable adhesion strength as is demonstrated of the 145 °C–20 min condition. Curing degree increases with the curing temperature to a limit beyond which the curing level is slightly reduced. Curing temperature over this limit can lead to bubble formation. Unknown chemical reactions may occur and additives will be released generating more volatile and bubbles. Adhesion strength is reduced significantly through the creation of voids and imperfect interfaces. Therefore, curing temperature higher than this limit should be avoided by all means.

## 5. Conclusions

The lamination process is arguably the most important factor that influences the durability of the polymeric multi-layer encapsulant of PV modules. In this study, the influences of the lamination conditions on the performance of the encapsulation system of PV modules has been investigated, considering both initial quality, and long-term stability. The performance of the encapsulation system is evaluated based on a number of different properties including bubble formation, crosslinking degree of EVA, chemical and optical stability, and adhesive strength at different interfaces. It is shown that lamination conditions resulting in comparable initial quality doesn’t guarantee equivalent long-term stability. Furthermore, it is evident that lamination conditions leading to similar characteristics in one of these properties alone cannot always ensure similar durability of the other properties.

Several degradation modes related to the encapsulation system are identified in this study, i.e., moisture ingress and EVA hydrolysis, discoloration, and loss of adhesion strength. The degradation of adhesion strength appears to be the most significant and most rapid degradation among these modes.

This study provides further understanding on the influences of curing degree of EVA on other properties of the encapsulation system which are not fully understood so far. In general, the stability improves with increasing curing degree up to a critical point. Curing beyond the critical point is detrimental to the stability of the encapsulation system and may result in the formation of voids, more transmittance reduction, more yellowness of EVA and quick adhesion degradation under thermal stress. Current guidance does not identify the maximum level and to some extent the industry follows a ‘more is better’ approach. This somewhat endangers long-term performance of PV modules. Therefore, there exists a window of gel contents that can lead to optimal general encapsulation durability. The optimal window of gel content for materials investigated in this study should be in the range of 84–90% that are cured between 135 °C for 10 min and 145 °C for 10 min. Laminates with gel content below 70% demonstrates low chemical and optical stability, weak adhesion strength at EVA-backsheet interface, and EVA flowing. Laminates with gel contents higher than 92% are more likely to become yellowness and are less stable in adhesion. To achieve the best performance of the laminates, EVA should be cured within the optimal curing range. In addition, too high temperature should be avoided to prevent the formation of bubbles. Too long curing time should also be avoided of over-curing while adequate curing time should be assured to establish sufficient adhesion strength at the glass-EVA interface.

The different reliability levels of the encapsulation system caused by the different lamination condition will influence the PV module performance. The losses in transmittance of 2–13% are identified during damp-heat exposure for the investigated lamination conditions, which would cause an energy yield difference of nearly the same amount. This leads to significant differences in terms of financial benefits of the operating systems. Other properties such as the chemical stability and the stability of adhesion strength also affect the performance of PV modules manifesting in corrosion, delamination and elevated leakage current.

Stability tests in this study are based on standard tests used in industry. Outdoor exposure will be different as stresses are not steady and will interact with each other. These standard tests aim to increase product quality beyond certification, which may not represent fielded modules’ real-life operation. Independent of these undeniable limitations of the standard certification tests, the application of these tests has yielded significant improvements in the performance of fielded modules and has near-eradicated certain failure mechanisms, which dominated in the past.

## Figures and Tables

**Table 1:** Summary of the performance of the laminates cured at different conditions.

Condi-tions	Gel con-tent (%)	Bubble Forma-tion	Chemical Stability		Optical Stability		Adhesion (N/cm)			
			O-H Peak Area after 1000 h Exposure	C=O Peak Area Decrease after 1000 h Exposure	Integrated T% Losses after 1000 h Expo-sure	YI Increase after 1000 h Expo-Sure	Glass-EVA (90° Peel)			EVA-Backsheet (T-Peel)
							Initial	Damp-Heat after 1000 h Expo-Sure	Thermal Cycling after 200 Cycles	
160 °C-10 min	88.9 ± 0.2	Yes	–	–	–	–	–	–	–	–
155 °C-10 min	88.5 ± 0.1	Yes	–	–	–	–	–	–	–	–
150 °C-10 min	87.5 ± 0.2	NM	–	–	–	–	3 ± 1	–	–	–
145 °C-20 min	92.0 ± 0.3	NM	4.2 ± 0.8	19% ± 1.2%	5% ± 1.3%	677% ± 89%	90 ± 18	12 ± 7	7 ± 2	43 ± 5
145 °C-10 min	90.6 ± 0.3	NM	4.4 ± 4.7	11% ± 3.1%	2% ± 0.9%	234% ± 76%	95 ± 16	8 ± 0.8	55 ± 11	35 ± 1
135 °C-10 min	83.9 ± 0.7	NM	4.2 ± 2.7	10% ± 1.6%	4% ± 0.6%	206% ± 93%	86 ± 28	12 ± 4	44 ± 14	21 ± 1
145 °C-5 min	80.6 ± 0.9	NM	7.7 ± 1.7	14% ± 1.9%	6% ± 1.8%	626% ± 71%	62 ± 23	15 ± 6	40 ± 15	19 ± 2
125 °C-10 min	68.0 ± 4.2	NM	10.6 ± 0.8	16% ± 2.3%	13% ± 3.9%	521% ± 127%	84 ± 18	27 ± 10	54 ± 13	7 ± 2

(Note: NM stands for not measurable).

## Data Availability

The data presented in this study are available on request from the corresponding author.
